# The genetics of protein S deficiency: unresolved questions and new leads

**DOI:** 10.1016/j.rpth.2026.106643

**Published:** 2026-05-12

**Authors:** Pablo García de Frutos

**Affiliations:** 1 Department of Molecular and Cellular Biomedicine, Institute of Biomedical Research of Barcelona (IIBB), Consejo Superior de Investigaciones Científicas (CSIC), Barcelona, Spain; 2 Department of Hemotherapy-Hemostasis, Institut d’Investigacions Biomèdiques August Pi i Sunyer (IDIBAPS), Barcelona, Spain; 3 Department of Atherosclerotic and Cardiovascular Disease Program, Centro de Investigación Biomédica en Red en Enfermedades Cardiovasculares (CIBERCV), Madrid, Spain; 4 Department of Mechanisms of Tumorigenesis and Tumor Progression, Unidad Asociada Institut de Recerca Hospital del Mar (IMIM)/Institut d'Investigacions Biomèdiques de Barcelona (IIBB)-CSIC, Barcelona, Spain

Protein S deficiency (PSD) was recognized as a cause of venous thromboembolism in the early 1980s. Since then, PSD has been a paradigmatic example of how disruption of endogenous anticoagulant pathways can confer a strong hereditary predisposition to thrombosis [1,2]. Together with protein C and antithrombin deficiency, it constitutes the group of high-risk thrombophilias caused by “inhibitor defects,” which provided the first mechanistic link between inherited molecular abnormalities and clinical disease. Yet even today, PSD remains the most diagnostically challenging of these conditions. Although new genetic data have brought valuable insights, the relationship between genotype and clinical phenotype remains complex.

The diagnostic complexity of PSD is rooted in the biology of the protein itself [3,4]. Protein S functionality is highly sensitive to exogenous influences; oral contraceptives, pregnancy, acute illness (including COVID-19), and liver dysfunction all can produce transient reductions that mimic hereditary deficiency. Moreover, protein S circulates largely bound to C4b-binding protein (C4BP), a complement regulator, an interaction that further complicates the interpretation of plasma measurements [5]. A further layer of complexity arises from enzymatic modification of protein S, a largely underrecognized phenomenon with potential impact on both diagnostic accuracy and the clinical utility of pooled plasma products [6]. These biological intricacies are well exemplified by a recent large population-based study that has established the prevalence of PSD with greater precision than ever before [7]. Strikingly, fewer than 10% of individuals with protein S levels in the deficient range carried a deleterious mutation in PROS1. When a causative mutation was present, however, thrombotic risk was substantially elevated, particularly for loss-of-function variants, which, though rare (approximately 1 in 10,000), conferred the highest risk. Milder mutations, found in approximately 1 in 500 individuals, were associated with a significantly lower thrombotic risk. Perhaps most intriguingly, low protein S levels carried a similar thrombotic risk regardless of whether an underlying mutation was identified. All these findings challenge simple genotype-driven risk stratification and underscore the clinical relevance of the phenotype itself.

The study of thrombophilia has been greatly facilitated by next-generation sequencing, which have expanded mutation detection even in patients without a classic thrombophilia profile [8,9]. This approach is exemplified by Bartylla et al. [10], who performed systematic sequencing of the entire PROS1 coding region and adjacent intronic boundaries in 276 patients with suspected hereditary PSD. The authors identified 48 distinct mutations in 101 patients, including 10 genuinely novel variants. These novel variants were evaluated using bioinformatic prediction tools and crossreferenced with protein S activity levels, providing a structured framework for the classification of PROS1 mutations.

The challenge of interpreting PROS1 variants is longstanding, but the tools available to address it have changed profoundly. Early studies on the molecular basis of PSD relied primarily on evolutionary conservation and basic structural modeling. Over the intervening decades, this landscape has been transformed considerably: structure prediction tools combined with population-scale allele frequency data now allow far more nuanced and accurate predictions of the variant effect. The present study by Bartylla et al. [10] operates within this richer analytical environment, and its application of contemporary prediction tools to novel PROS1 variants represents a meaningful methodological advance. That said, the authors are appropriately cautious emphasizing the need for comprehensive, multiparameter variant evaluation [3]. In suspected hereditary PSD, a causative mutation is identified in less than half of the cases, a diagnostic gap that remains incompletely understood. Several studies have identified large deletions and duplications in up to half of those unexplained cases [11,12]—structural variants that would not be detected by the sequencing approach employed by Bartylla et al. [10]. Some authors have attributed this elevated frequency of structural rearrangements to the specific genomic architecture of PROS1, which appears particularly prone to copy number variation [13].

An instructive illustration of how mutation type shapes clinical severity is provided by the authors’ findings on protein S Heerlen, a well-characterized variant identified in approximately 20% of mutation-positive cases in this cohort—a frequency consistent with previous studies. Notably, Bartylla et al. [10] report a high prevalence of concurrent factor V Leiden (FVL) carriage among protein S Heerlen individuals (5 of 24). The coexistence of PSD with FVL has been proposed to confer a synergistic, rather than merely additive, increase in thrombotic risk [14]. This could reflect the mechanistic convergence of 2 defects within the same anticoagulant pathway: when both the cofactor (protein S) and its substrate (factor Va, rendered APC-resistant by FVL) are impaired, the regulatory circuit is doubly compromised. This concept of thrombophilia as a multigenic disease implies that individuals carrying newly described PROS1 variants of modest individual penetrance may develop thrombosis primarily when additional genetic risk factors coexist.

Taken together, this study contributes to an evolving paradigm shift in thrombophilia research: away from binary classifications of “deficient” vs “normal” and toward a variant-specific, context-dependent framework for risk stratification. The detailed functional and structural mapping of variant locations, the transparent discussion of methodological limitations, and the careful attention to prevalence data all strengthen the study’s utility as a reference resource. The field now requires prospective, population-based data to determine the true thrombotic penetrance of newly identified PROS1 variants across diverse genetic and environmental backgrounds. In the interim, the message from this study is clear: in suspected hereditary PSD, clinical and laboratory assessment must remain comprehensive, genetic testing must be interpreted within its functional context, and novel variants must be cataloged rigorously for genotype–phenotype correlations to be useful in the clinical practice [1,6]. It is also worth noting that among patients in whom no thrombophilic trait is identified by standard clinical workup, a significant proportion of them are found on broader genetic testing to carry variants in thrombophilia-associated genes, including prominently variants of uncertain significance, pointing to a more complex and incompletely defined relationship between genotype and thrombotic risk [15].

Finally, it is worth to consider the broader biological significance of protein S that has come to light since its discovery. Protein S is not solely an anticoagulant; it also functions as a ligand for membrane receptors (TYRO3, AXL, and MERTK), acting in concert with GAS6 to promote efferocytosis, the phagocytic clearance of apoptotic cells, and to modulate innate immune responses dampening excessive inflammation. These nonanticoagulant functions have gained considerable clinical relevance in recent years; the SARS-CoV-2 pandemic brought them into especially sharp focus [16,17]. Emerging evidence on the local role of protein S in the context of infection highlights its properties in regulating immunothrombosis and carries broader implications for how protein S function should be studied and interpreted [18]. A PROS1 variant that produces only mild anticoagulant impairment may nevertheless perturb TAM receptor signaling and dysregulate immunothrombosis in ways that current functional assays do not capture. As the catalog of PROS1 variants expands, the field will need to ask not only whether a given mutation impairs anticoagulation but whether it disrupts the full repertoire of protein S functions.
